# ProteomeScout: a repository and analysis resource for post-translational modifications and proteins

**DOI:** 10.1093/nar/gku1154

**Published:** 2014-11-20

**Authors:** Matthew K. Matlock, Alex S. Holehouse, Kristen M. Naegle

**Affiliations:** Department of Biomedical Engineering and the Center for Biological Systems Engineering, Washington University, St Louis, MO 63130, USA

## Abstract

ProteomeScout (https://proteomescout.wustl.edu) is a resource for the study of proteins and their post-translational modifications (PTMs) consisting of a database of PTMs, a repository for experimental data, an analysis suite for PTM experiments, and a tool for visualizing the relationships between complex protein annotations. The PTM database is a compendium of public PTM data, coupled with user-uploaded experimental data. ProteomeScout provides analysis tools for experimental datasets, including summary views and subset selection, which can identify relationships within subsets of data by testing for statistically significant enrichment of protein annotations. Protein annotations are incorporated in the ProteomeScout database from external resources and include terms such as Gene Ontology annotations, domains, secondary structure and non-synonymous polymorphisms. These annotations are available in the database download, in the analysis tools and in the protein viewer. The protein viewer allows for the simultaneous visualization of annotations in an interactive web graphic, which can be exported in Scalable Vector Graphics (SVG) format. Finally, quantitative data measurements associated with public experiments are also easily viewable within protein records, allowing researchers to see how PTMs change across different contexts. ProteomeScout should prove useful for protein researchers and should benefit the proteomics community by providing a stable repository for PTM experiments.

## INTRODUCTION

ProteomeScout is a database of protein post-translational modifications, protein annotations and a web resource for analysis of PTMs and proteins. ProteomeScout is unique in the world of post-translational modification (PTM) databases—it is the only database that encompasses PTM compendia and allows for the community deposition of PTM experiments, and it is the only web service that has built-in subset selection and enrichment for exploring and analyzing PTM experiments. ProteomeScout is also unique in the world of protein resources—it is a resource that entails a flexible, track-based viewer of protein annotations. The purposeful design of these features was inspired by the historical progression of resources for other types of molecules, including: the UCSC Genome Browser (1) and the Gene Expression Omnibus (GEO) (2). The rapid expansion in the identification of protein PTMs, enabled by high-throughput experimental techniques (3), has led to explosive growth in the number of specialized PTM databases, although none of which reflect the full capabilities of the UCSC Genome Browser or the GEO. A sampling of PTM-specific databases includes Phosida (4), PhosphoSitePlus (5), phospho.ELM (6), PTMScout (7), dbPTM (8), PTM-SD (9), PTMCode (10) and O-GLYCBASE (11). The names of these databases reflect the progression of PTM research, and despite initially being focused on phosphorylation, many of them now encompass multiple modification types.

Growth in PTM identification is just one part of the rapid expansion in PTM-centric research; the other part involves the quantitative profiling of PTM changes across conditions. New experimental capabilities have enabled a number of studies, including high-throughput profiling of tyrosine phosphorylation changes in response to receptor tyrosine kinase activation (12,13), methylation response to methyltransferase inhibition (14) and ubiquitination response to proteasome inhibition (15). These datasets contain rich information about the regulation of proteins. However, supplementary data standards in this research field have made it difficult for researchers to garner the power of this information.

The *status quo* for PTM data involves publication of isolated, static supplementary tables. This means that there is no single standard format, no central repository and, due to the volatility of protein databases (16), there is no persistent mapping of identified PTMs to protein database records. Protein identifiers become out-of-date relatively quickly, and in the most extreme cases, disappear altogether—as with the discontinuation of the International Protein Index (IPI) identifiers. The desire to have a stable, useable and accessible repository of experiments has happened before—for high-throughput gene expression experiments. This desire resulted in the development of the GEO (2), now an important mainstay in the research community. We have created ProteomeScout to serve as a living repository of high-throughput PTM experiments, thereby enabling the stability and accessibility of published experimental data in the hopes that in the future it may have as much utility in the field of PTM research as GEO does in gene expression research. To encourage its adoption as a standard repository, we have focused on making it easy to deposit datasets and created the ability to maintain private datasets, which enables private analysis and evaluation prior to publishing to the public domain.

In addition to a centralized repository, useful analysis methods are also necessary, in order to dissect meaningful information from high-throughput PTM experiments. We have developed a number of methods, and utilized them in our own work, which led to novel insights from PTM experiments, including prediction of novel protein interactions from dynamic phosphorylation data (17), the identification of upregulated motifs downstream of the the EGFRvIII receptor (18), prediction of PTMs acting in specific pathways (19) and dissection of poorly understood signaling networks (20). ProteomeScout incorporates a number of these analysis tools, plus others, with a focus on ease-of-use, flexibility and reproducibility.

Another tool needed in protein-centric research is a flexible protein viewer, which allows for the simultaneous visualization of complex protein annotations. PTMs are just one complexity in proteins, which are ultimately biochemical machines. It is a difficult task to predict how alterations in sequence properties, such as those introduced by modifications or through non-synonymous polymorphisms, will affect a protein's structure and function. Chromosomes are also complex linear sequences composed of regulatory elements, modification sites, exons, introns and so forth. The UCSC Genome Browser was developed to give researchers the ability to visualize the components that make up chromosomes. Proteins, composed of complex secondary structure elements, independent domains, sequence variation, PTM sites and other linear components, beg for a similar visualization method. The UCSC Genome Browser developers responded to this need and created the Proteome Browser (21), which appears to be no longer supported (i.e. the resource is no longer available from the Genome Browser and was last referenced in an update in 2011 (1,22)). DASty (23) also provides such a resource. However, with the extent of PTM modifications that can occur on a protein special PTM-centric viewer features might be additional required. These features might include the use of different colors for different residue types, filtering and the ability to zoom in on particular PTMs or protein features. ProteomeScout includes a modern, web-based visualization tool for proteins inspired by the ideas behind the UCSC Genome Browser and DASty, which includes features that are particularly well-suited for visualization of complex PTMs and the dissemination of this information by exporting the viewer using Scalable Vector Graphics formats.

**Figure 1.**
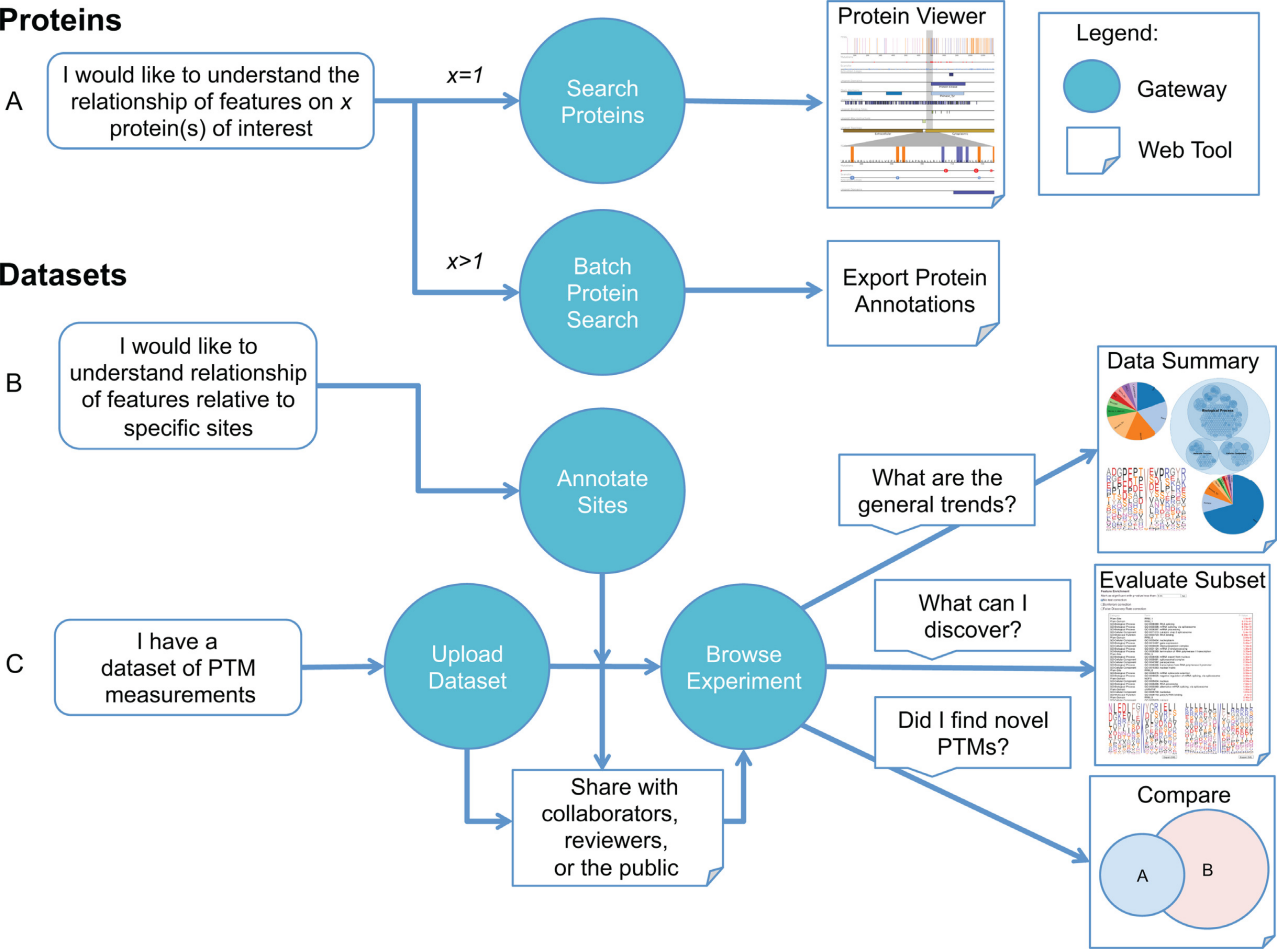
A map of ProteomeScout features and example user workflows. Gateway refers to the homepage menu items and Web Tool refers to actions that occur on subpages off of the main menu. (**A**) Methods for searching and interacting with proteins. (**B**) Tools for understanding features around specific protein residues. (**C**) Methods for loading and evaluating datasets of PTMs.

This paper will discuss ProteomeScout features and the underlying methods. We have included Figure 1 as a reference map for the type of interactions a typical user might have. We will focus on those features that make ProteomeScout most unique. These include the breadth of PTMs, the repository aspects for PTM experiments, the analysis suite available for datasets and the protein viewer for visualization of the relationship between complex protein annotations. ProteomeScout can be found at https://proteomescout.wustl.edu.

## MATERIALS AND METHODS

### Protein annotations

The protein loading process begins when accessions are loaded through one of the following three interfaces: (i) *Dataset Upload*, (ii) *Batch Search* or (iii) *Annotate Sites*. When a protein accession is passed to ProteomeScout through these methods, the appropriate external database is queried for the taxonomy and protein sequence associated with that accession. If the sequence from that species already exists in ProteomeScout, then the accession is added. If the protein sequence does not already exist, then all of the following protein annotations (Table 1) are added to ProteomeScout for the new protein.

**Table 1.** Protein annotations and their sources currently incorporated in ProteomeScout

Annotation	Source
Domains	Pfam (27), UniProtKB (31)
Structure	UniProtKB (31)
Binding sites	UniProtKB (31)
Macrostructure	UniProtKB (31)
Topology	UniProtKB (31)
Gene Ontology terms	Gene Ontology Consortium (38)
Non-synonymous polymorphisms	dbSNP (30), UniProtKB (31), NCBI (24)
Gene expression	GNF SymAtlas (39)
Database identifiers	UniProtKB/SwissProt (31), NCBI (24), GenBank (32), PDB (33)
Kinase predictions	Scansite (29)
SH2 and PTB binding predictions	Scansite (29)
Kinase activation loops	ProteomeScout Analysis (7)

#### Protein sequences

Proteins specified by UniProt accession numbers are obtained from the UniProtKB batch query web service. Proteins specified using other accession numbers are, if possible, fetched from NCBI Entrez (24) using the Biopython (25) Entrez API.

#### PTM ontologies and rules

PTM ontologies are established from the UniProt ptmlist (http://www.uniprot.org/docs/ptmlist) and the ExPASy FindMod tool (http://web.expasy.org/findmod/findmod_masses.html) (26). Rules for a PTM are established by extracting the ‘Target Residue’ and ‘Taxonomic Range’ from the UniProt ptmlist. These rules are applied when modified peptides are loaded from *Upload Dataset* —ProteomeScout ensures that the annotated residue and the species of the designated protein accession match the ‘Target Residue’ and ‘Taxonomic Range’ of the modification.

#### Pfam domain predictions

When proteins can be cross-referenced or are queried with a UniProt accession, pre-generated domain predictions from the Pfam Web service are fetched (27). In the event that the protein sequence cannot be cross-referenced to a UniProt accession, the Pfam sequence search is used to retrieve new domain predictions.

#### GO annotations

Annotations of ‘Biological Process’, ‘Molecular Function’ and ‘Cellular Compartment’ are obtained for each protein record by querying the EBI QuickGO (28) service, using the accession number provided by the user query.

#### Scansite predictions

Scansite predictions are retrieved from Scansite 3.0 (29), available at http://scansite3.mit.edu, using the proteinScan tool. The stringency value is set to ‘low’ to retrieve all available predictions. The motifClass is chosen based on the sequence's taxonomic lineage. Any sequence with taxonomic node ’mammalia’ is queried with the ‘MAMMALIAN’ motifClass, while sequences with taxonomic nodes ‘Sacchromycotina’ or ‘Saccharomyces’ is queried with the ‘YEAST’ motifClass. Any protein not satisfying these taxonomic criteria are excluded from Scansite predictions.

#### Non-synonymous polymorphisms

Protein records obtained from NCBI are parsed for region annotations containing the ‘variant’ region name field. Protein records obtained from UniProtKB are parsed to extract ‘Natural Variant’ annotations. Variants representing single amino acid changes from either of these sources are stored in ProteomeScout, along with associated dbSNP (30) accessions, if present, and dbSNP is then queried to retrieve the ’Clinical Significance’ feature of the SNP. ‘Clinical Significance’ takes one of the following discrete values: pathogenic, non-pathogenic, unknown, other, untested, probable-non-pathogenic, probable-pathogenic and drug-response.

#### Alternative accessions

Accession numbers provided in a user query are cross-referenced to obtain accession numbers for UniProt/SwissProt (31), NCBI (24), GenBank (32), PDB (33) and gene synonyms using the Protein Identifier Cross Reference (PICR) service from EBI (34).

#### Taxonomic range

The complete taxonomic ranges for all species are parsed from the NCBI or UniProtKB record. In the event that there is a host organism (for viral proteins) the taxonomic lineage for the host organism is obtained from the Entrez taxonomy web interface using the Biopython Entrez API (25).

### Implementation

ProteomeScout is written in the Python programming language using the Pyramid Web Application Development Framework. Interactive user interface elements are implemented in HTML, CSS and JavaScript, using the JQuery, JQuery UI and D3 libraries. All proteomic data is stored in a MySQL relational database, which is accessed through Python using the SQLAlchemy Object Relational Mapping API. The web application is deployed on an Ubuntu Linux Server (v12.04) using the Apache Web Server with modwsgi. ProteomeScout uses the Python Celery parallel computing toolkit to run distributed data import, export and statistical testing over multiple threads and machines. ProteomeScout uses JavaScript and the D3 library for visualization of data in the protein viewer, experiment summary/explorer and the database statistics pages. Venn diagrams are generated with Ben Frederickson's D3-based Venn Diagram tool. ProteomeScout's documentation, ticketing system and Mercurial repository are hosted freely by Assembla as an open-source project; available at https://www.assembla.com/spaces/proteomescout.

### Data submission

Data submission is done interactively on the web site. The first step in dataset loading is data file preparation. Users should use a text or Excel editor to create a tab-separated data file containing the appropriate headers that designate the columns of interest (protein accession, peptide sequence or site number, modification type and, if applicable, data columns). In the second step, a registered user uploads this file to ProteomeScout. The remaining steps will walk users through verifying the column assignments, checking a subset for potential problems and setting the metadata (such as a description, experiment title and conditions of the experiment). If published, a PMID (PubMed identifier) can be used to automatically retrieve title, authors and other journal information. Finally, after the dataset load is completed, owners will receive an email notification and link to their dataset's error log. Errors are described in the log and an easy process exists for downloading the subset of the data file with errors and then reloading the corrections. Example screen shots and files are provided on the help documentation under the title ‘Loading a Dataset’.

### Licensing and downloads

ProteomeScout code and graphics are licensed under a Creative Commons Share-Alike with Attribution 4.0 International license. The ProteomeScout database download additionally requires ‘non-commercial’ use, since a majority of the compendia require that restriction. Registration is required for the resources that involve assignment of ownership (*Upload Dataset, Annotate Sites*) or result in the email communication of results (*Batch Search, Evaluate Subsets*). *Protein Viewer, Statistics and Dataset Download* do not require user login. There are no restrictions for registering for an account, except a valid email address.

### Analysis methods

#### Evaluate Subsets

The *Evaluate Subsets* feature of ProteomeScout allows users to create dataset subsets based a set of logical criteria. Here, a dataset refers to either an ‘experimental’ dataset or an ‘annotate sites’ dataset. To create a subset, users may use the interactive query tool on ProteomeScout or load custom definitions using a flat-text file format. From the interactive query tool, users have access to protein annotations, quantitative data and peptide sequences. Example queries are given in Table 2.

**Table 2.** Query categories available in *Evaluate Subsets*, with example queries

**Query category**	**Example application**	**Query language built in ProteomeScout**
Quantitative data	Two-fold increase in first minute	Time_1 (min)/Time_0 (min) >= 2
	and decreasing by second minute	**AND** Time_2 (min) < Time_1 (min)
Metadata: categorical data (Table 1)	Proteins in nucleus	GO: cellular compartment = ‘Nucleus’
Subset: in or not-in a previously saved subset	Proteins not in nucleus	**not in** ‘nucleus subset’
Cluster: user imported cluster definitions	Peptides in first and second clusters	cluster ID = 1 **OR** cluster ID = 2
Sequence: regular expression search of peptides	Peptides matching SHC's PTB binding motif	NP.y

Testing for enrichment of labels in a subset is based on over-representation of a label within the subset, relative to a background and compared to random expectation (Fisher's exact test). Users can use the entire dataset as a background or specify a subset of the data to use as a background. Feature enrichment is performed over all GO terms, Scansite predictions, Pfam predictions and kinase activation loop predictions using the Fisher's exact test. Fisher tests are evaluated using the NumPy and SciPy Python packages (35). Motif enrichment is performed using the greedy search algorithm described in (18) with a branch cutoff value of 0.01 and a user-defined sequence length. Bonferroni and False Discovery Rate (36) multiple hypothesis correction techniques are also available as options for users.

#### MCAM

Support for MCAM, a method of ensemble clustering with feature selection (19), has been integrated in the *Evaluate Subsets* tool. Users can download annotated versions of their data using the *Export Experiment* tool, cluster using MCAM, import their clustering annotations, and perform enrichment analysis across all clusters and all cluster sets. Feature selection and evaluation can then be performed using the enrichment results, which ProteomeScout sends by email. The MCAM Mercurial repository is also hosted by Assembla and linked to the ProteomeScout Assembla page.

#### Compare Datasets

The *Compare Datasets* tool allows users to compare the modified residues identified in their experiments with other experiments and compendia. We consider a modified residue to be shared between two experiments if three conditions hold. First, the modified residue is assigned to the same protein. Second, the modified residue is at the same position in the protein. Third, the modified residue is annotated with the same modification type.

#### Report Ambiguity

The majority of high-throughput measurements of PTMs involves mass spectrometry-based identification of protein fragments. Consequently, there is often ambiguity regarding which protein or proteins, a peptide has originated from. The ProteomeScout web interface highlights when a peptide ambiguously maps to an assigned protein sequence by searching for alternate matching proteins in the ProteomeScout and UniProtKB databases. Ambiguously assigned peptides can easily be reassigned to alternate sequences via a drop-down menu on the *Report Ambiguity* tool. We also provide a tool to assign all ambiguous peptides to ‘default’ proteins using the *Assign Defaults* feature. ‘Default’ proteins are chosen as the protein with the most reported modifications in ProteomeScout. This tool allows users to create a new dataset representing assignments of peptides to ‘canonical’ protein records, which is particularly useful for evaluating the novelty of modified residues using the *Compare Datasets* tool.

#### Kinase activation loops

Kinase activation loop predictions are based on finding the amino acid sequence, spanning less than 35 amino acids, between the N-terminal ‘D(F/P/L/Y)G’ and C-terminal ‘(A/S/P)(P/I/L/W)(E/D)’ flanking sequences. Identification of the degenerate flanking sequences was defined in the PTMScout publication (7).

## RESULTS

### ProteomeScout overview

**Figure 2.**
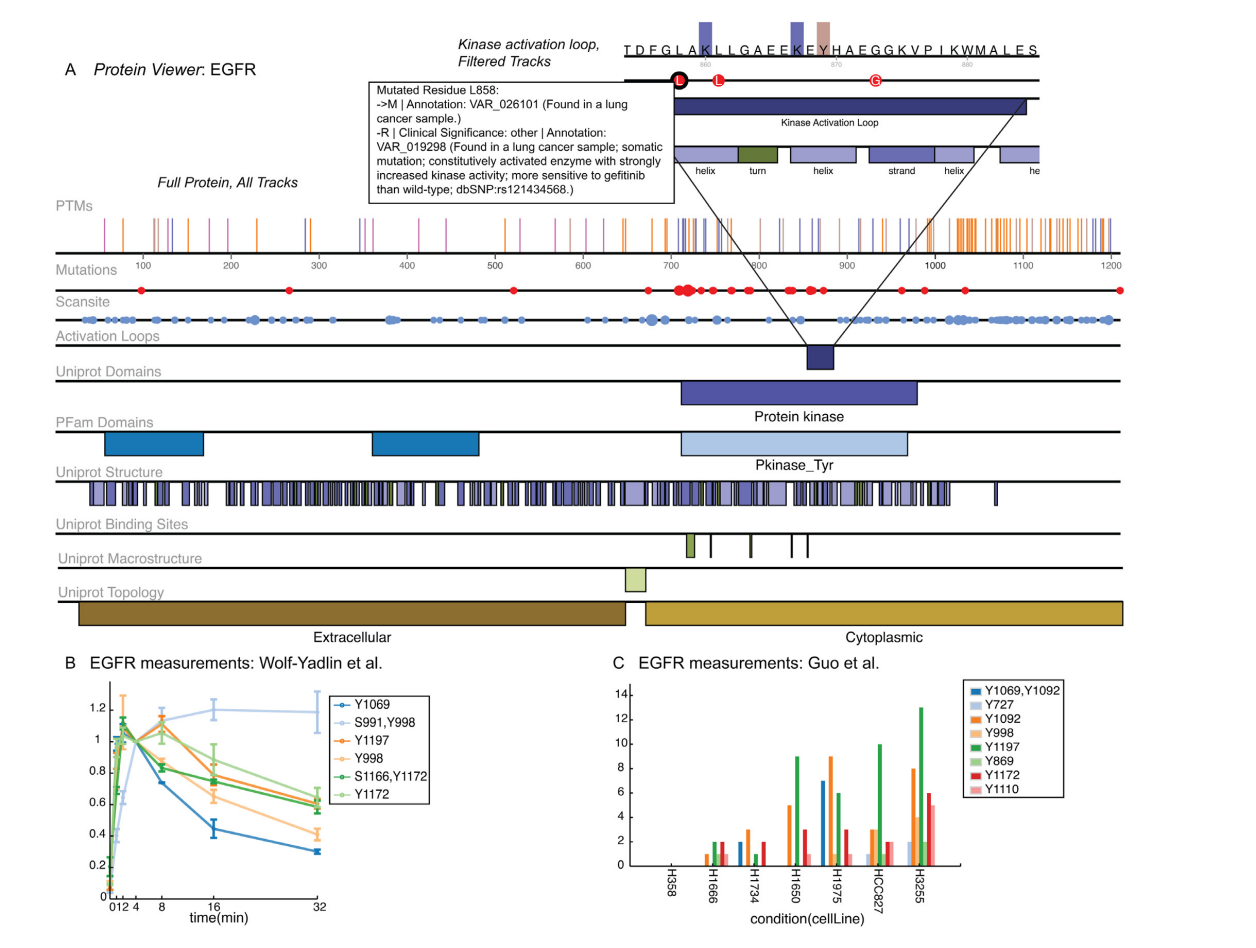
Example of protein information available on ProteomeScout. All figures were exported directly from the ProteomeScout interface (using ‘SVG Export’). (**A**) The ProteomeScout view of the EGFR sequence, full track below with an inset, which is the expanded view of the kinase activation loop. PTM track: bars indicate where PTMs occur, the color indicates the type of residue and, when applicable, the bar height is proportional to the number of different documented modifications. Mutations track: size of circle is proportional to the number of documented non-synonymous polymorphisms. Scansite track: Size of circle is proportional to the number of Scansite predictions. Labels under annotations are suppressed when the annotation is small, relative to the label. Labels are available when hovered over by the cursor or when that area is expanded; for example, the annotations for the L858 mutation are shown (inset). (**B**) Quantitative data for measurements from (13) experiment measuring relative phosphorylation as a function of time following EGF stimulation of human mammary epithelial cells. (**C**) Quantitative data for measurements from (40) experiment measuring phosphopeptide counts across a number of lung cancer cell lines.

The ProteomeScout database is built around two central data types: datasets and proteins. Experimental datasets encompass sets of modified peptides, which are associated with a specific protein record and may be associated with quantitative data. Protein records are associated with annotations (Table 1). ProteomeScout has analysis features specific to datasets (Figure 1B) and specific to proteins (Figure 1A), detailed below. A shared feature across many pages of ProteomeScout appears as a button labeled ‘Export SVG’. This button is associated with graphics generated on ProteomeScout and indicates that graphic can be downloaded in SVG format. The SVG format ensures that the figure is sufficient for both screen and print resolution. Additionally, it allows for the easy manipulation of the graphic; for example, fonts can be individually scaled in a graphics editor. This feature was designed to enable the easy incorporation of figures in written and oral communications and we have made ample use of it here (Figures 1 and 2).

With an emphasis on community-driven deposition, the coverage of PTMs within ProteomeScout will continue to expand. ProteomeScout currently includes six major curated sources of PTMs, which are updated frequently, (UniProtKB (31) , PhosphositePlus (5), Phospho.ELM (6), HPRD (37), dbPTM 3.0 (8) and O-GLYCBASE (11)) and 77 experiments, 17 of which are currently public. These publicly available experiments, which were primarily loaded by the developers of ProteomeScout from published experiments based on an overlapping interest in the biological questions of the lab, contain 144,181 pieces of quantitative data, where 19,515 modified peptides are associated with at least one quantitative measurement. The large number of private experiments, from across the globe, indicates researchers are actively utilizing ProteomeScout as a starting point for further analysis as none of these experiments reflect data contributed directly by the developers of ProteomeScout. Combined, the compendia and public experiments include 420, 000 sites of modifications, which cover 250 unique modification types from 1,305 species. The database is capable of handling 366 total modification types, based on the controlled vocabulary defined by UniProt (31). Current statistics are available from the ProteomeScout *Statistics* page (linked from the home page). These statistics indicate that the majority of documented PTMs come from human, mouse and rat proteins. The top five most annotated modification types are: phosphorylation of serine, threonine and tyrosines; and acetylation and ubiquitination of lysines. ProteomeScout data is available for download, in bulk or as species—and modification-specific files. These flat-text files, which contain coverage of PTMs from multiple compendia and public experiments—with related protein annotations—will be especially useful for those groups exploring proteome-wide phenomena of PTMs.

### Datasets

Experimental PTM datasets are created when users upload data to ProteomeScout *via Upload Dataset* (Figure 1C). *Upload Dataset* requires a tab-separated file with at least three columns and their associated column headers, designating the protein accession, the modified peptide or protein residue and the type of modification. Columns of quantitative data, with header labels identifying their data type, can also be included. Data replicates can be included separately, with different ‘experimental runs’ indicated, or as an average with standard deviations (Figure 2B). *Upload Dataset* walks users through the upload process. When loading is finished, the dataset is private for the owner, unless they choose to either *Share* the dataset, with collaborators or reviewers, or make the dataset available to the public (*Publish*). Data associated with public experiments becomes visible on individual protein pages (Figure 2B and C), is included in the database download, and becomes available for public analysis.

### Annotate sites

*Annotate Sites* (Figure 1B) is a ProteomeScout feature that allows users to explore protein positions, in the same way as experimental data. However, it does not require that these positions be sites of modifications. For example, they could instead be sites of identified SNPs from patient samples. *Annotate Sites* has the same process for loading, sharing and publishing, as well as access to dataset analysis tools (discussed below). This feature could be utilized for a variety of reasons. For example, for a set of clinical SNPs, one could explore where these occur relative to secondary structure elements, known modifications, or protein domains, which has the potential to improve the prediction of whether a SNP affects protein function or regulation.

### Dataset analysis

There are several powerful analysis tools available for datasets, which include at-a-glance summaries of constituting protein annotations, subset creation with enrichment testing and comparison with other datasets, including identifying novel modifications. These tools were developed to assist researchers in discovering important biological information within datasets. Here, we will focus on analysis tools that allow users to explore the composition of proteins and peptides in their datasets and test the statistical significance in subsets of their datasets. Other analysis tools, such as *Dataset Comparison* and *Ambiguity Reporting*, are discussed in the ‘Materials and Methods’ section.

### Data Summary

The *Data Summary* panel of Figure 1C provides example of charts and motif logos created by ProteomeScout, which summarize the composition of the proteins and peptides in a dataset. We anticipate that a global view of dataset composition will be helpful for understanding the relationship between PTMs and cellular processes and that motif logos may indicate a global view of the presence of regulatory sequences or systematic biases. We use nested, circular graphs for depicting GO terms, in order to capture the hierarchical nature of these annotations. These graphs can be expanded to see the nested characteristic and relative number of instances of any particular term. All summary graphs and logos can be exported using the ‘Export SVG’ feature. Users can automatically select all proteins or peptides matching a certain criterion in any of the categories as a subset from this page, which will take them directly to the *Evaluate Subsets* feature. The *Evaluate Subsets* page will then list the subset of proteins and peptides, and their annotations, as well as testing for enrichment of annotations in this subset, relative to the full dataset.

### Evaluate Subsets

We have created a *Subset Selection* tool, available from *Evaluate Subsets*, that allows users to create simple, or compound, logical selection criteria for creating a subset. The subset is then tested for enrichment in protein and peptide annotations, with enrichment thresholds controlled by the user, including the selection of appropriate multiple hypothesis correction methods (Bonferroni or False Discovery Rate (36)). Subsets can be saved and shared. For example, a user could create a subset based on peptides from proteins known to undergo nuclear localization, which they could then save as the ‘nucleus subset’ (Table 2). That saved subset could then become a background for enrichment testing for other subsets of the ‘nucleus subset’. As an example of how this feature might be useful, this subset may show enrichment in motifs, which might indicate shared regulatory enzymes that are specific to nuclear-localized proteins.

Should the user-interface for dataset subset selection be insufficient, users can export the dataset and assign their own ‘clusters’ for reimport and selection. This export/annotate/import feature can be used to interface with our MATLAB-based ensemble clustering software, MCAM (19), which we have used previously to predict new protein interactions from dynamic measurements of tyrosine phosphorylation (17). There are innumerable types of subsets that users can create through *Evaluate Subsets* and so we have included a set of examples for demonstration in Table 2. The flexibility and power of this interface should allow users to create subsets of any type and explore the composition of them with regard to their protein and peptide makeup.

### Proteins

ProteomeScout users can visualize the relationship between protein annotations in a track-based system using the *Protein Viewer* (Figure 2). A track maps annotations (e.g. Pfam domains) onto the protein sequence. Tracks of different annotation types are stacked vertically so that multiple annotation types can be viewed relative to each other (e.g. PTMs will be above Pfam domains). Users can hide tracks and filter PTMs from the PTM track based on contributing experiments and/or the type of modification. Finally, users can zoom-in on a section of protein sequence, using a click-drag-release across the area of interest. The zoomed view appears below the full sequence view and maintains any track filtering applied, as described previously. The zoomed-section can be switched by doing a new click-drag-release on the full track. The features of a track-based viewer, with the ability to zoom, allow researchers to co-visualize a large number of protein annotations—from the macro- to the micro-scale.

### Protein Viewer

Figure 2 demonstrates the track system, along with track filtering and zoom for an example protein, the epidermal growth factor receptor (EGFR, UniProt P00533). Figure 2A demonstrates the unfiltered track for EGFR, showing currently available tracks and modifications. The inset in Figure 2A shows just a subset of tracks and is zoomed-in on the kinase activation loop. Every track annotation has a mouse-over text box; we have included an example for EGFR's L858 mutations. This flexible track-based viewer of protein annotations will help researchers to understand the relationship between a large numbers of complex annotations. For instance in this example, users can easily see the relationship of the L858 mutation relative to known PTMs and secondary structure elements, while also seeing that the mutation is within the activation loop of the kinase catalytic domain. The SVG export feature will allow them to easily share these relationships with others in written or visual communications.

The *Protein Viewer* is the landing page for a selected protein in ProteomeScout. However, in addition to the viewer, proteins have a number of other webpages, which display: GO terms (38), cross-referenced database identifiers, gene expression (39) and any associated quantitative data from experiments. This particular EGFR record is associated with 54 GO terms, 6 Affymetrix array probes measuring expression across human tissues and the NCI60 panel of cell lines, 99 modified residues and quantitative data from six public experiments. *Protein Search* often returns multiple isoforms or multiple database records for a protein, which arises from the design of ProteomeScout to maintain the fidelity of published experimental datasets, which requires ProteomeScout to map to multiple, disconnected protein databases. We have found that, for most purposes, choosing the protein record with the most PTM annotations, which is clearly denoted on the search page, is often the most useful, since it usually demarcates the canonical isoform, which will have the most annotations of all types.

The unique aspect of a database with quantitative data measurements coupled with PTMs on proteins is demonstrated for EGFR with data from two public experiments, Figure 2B and C. The experiment in Figure 2B measured tyrosine phosphorylation in human mammary epithelial cells following stimulation with EGF (13) and includes data for six phosphopeptides, encompassing eight phosphorylation sites on EGFR. Because this experiment contained the average data values of triplicate measurements and the standard deviations, standard deviation bars are included on the line graph. For juxtaposition, Figure 2C shows measurements on EGFR from another experiment by Guo *et al.* (40) in a bar graph, since this experiment indicated measurements made across cell conditions instead of time. The experiment by Guo *et al.* profiled tyrosine phosphorylation in seven lung cancer cell lines, where quantification was based on the number of times a phosphopeptide was identified in an experiment (40). The coupling of protein records directly to quantitative measurements of PTMs could be a powerful tool for researchers. For example, Figure 2B and C demonstrates a number of EGFR phosphorylation sites that are immediately responsive to EGF and a number of which also occur in various lung cancer lines.

### Batch protein search

Finally, with regard to protein features, users can retrieve protein annotations from ProteomeScout using the *Batch Protein Search* (Figure 1A). This search takes a set of protein identifiers and returns, *via* email, a tab-separated file of proteins and their annotations. This file is in the same format as the full dataset download, which could be used directly for finding annotations of particular proteins. However, the *Batch Search* tool has the advantage of automatically searching for a particular protein identifier and loading it, or a protein record, if they do not already exist in ProteomeScout. Both ProteomeScout's dataset download and *Batch Search* results can be used for large-scale exploration of the relationships between proteins and protein annotations.

## DISCUSSION

ProteomeScout is a comprehensive PTM and protein resource. Perhaps the most forward-looking aspect of ProteomeScout is its role as a repository of high-throughput PTM experiments. Access to experimental datasets, as a whole, as well as linking quantitative measurements to individual proteins, will enable better community utilization of the rich source of information that is captured in high-throughput PTM experiments. We designed dataset permissions and sharing to encourage the following workflow in the scientific community: first, dataset upload and private analysis, including sharing with collaborators; second, inclusion of supplementary information in a publication and creation of a web-token for blind reviewers to evaluate the dataset; and finally, sharing of the dataset with the public, following manuscript publication. This process ensures the reproducibility of specific results and records, despite dynamic protein databases.

Fortunately, there are many wonderful curated sources of PTMs, such as PhosphoSitePlus (5), dbPTM (8) and UniProtKB (31), which have been incorporated into ProteomeScout. The combination of compendia with primary experiments makes the ProteomeScout database download a comprehensive—and constantly expanding—source of PTM knowledge. One drawback to the community-driven aspect is the possibility that unverified or poor-quality data could be added to the database. We guard against this possibility by: (i) requiring dataset owners to acknowledge terms of use, which specifically outlines the importance of adding high-quality data, (ii) providing the *Annotate Sites* feature, which allows users to explore unverified data in exactly the same ways as experiments and (iii) reserving the right as administrators to remove questionable data. Additionally, researchers could use the number of different experiments documenting a PTM as a proxy for the confidence associated with a PTM's identification. The number of experimental annotations are available in the database download, as well as on the website. We believe, despite the unique challenges of a community-driven resource of PTMs, that the advantages far outweigh the complications.

There are features in a number of PTM-centric resources that future versions of ProteomeScout will ideally incorporate, or link directly to. For example, UniprotKB (31) and PhosphoSitePlus (5) currently have the most descriptive annotations about the effect of PTMs on protein function, when the effect is known. Currently, ProteomeScout directly links to Uniprot records, but not PhosphoSitePlus. Also, it is clear that the general wealth of structural information within the PDB will be helpful for understanding the role of PTMs on regulating protein structure and function. Most notably, PTM-SD (9) curates the PDB for structures containing PTMs. Finally, there are several resources that are dedicated to inferring function of PTMs by combining proteome-level information with computational approaches. For example, PTMcode (10) documents and predicts functional relationships between PTMs based on calculations such as co-evolution. dbPTM (8) uses protein–protein interactions to infer potential PTM-specific interactions and associated motifs. ProteomeScout introduces the connection of quantitative measurements to PTM records and the visualization of PTMs relative to other protein features. To enable a future goal of improved linking and incorporation of specific features from existing resources in later versions of ProteomeScout, we have written ProteomeScout as an open-source project using agile software development methods. We hope this will enable community-driven contributions of protein and PTM features. For example, the developers of resources like dbPTM, could capitalize on the protein viewer software to add a track for highlighted domain–peptide interactions or surface accessibility predictions. Finally, we hope that this resource, which incorporates a number of existing compendia and experiments, may reduce the efforts required of researchers who must first curate PTM annotations prior to building novel methods for inferring PTM function.

In addition to the PTM-centric aspects of ProteomeScout, the more global protein-centric tools of ProteomeScout will be useful for a broad range of researchers. Specifically, the *Protein Viewer*, with track-based views of protein annotations, with the ability to zoom and export an editable graphics file, is different than other protein resources, even protein-viewer centric resources. EBI's DASty service (23) dynamically builds protein viewer tracks based on the active retrieval of information from DAS-enabled databases. However, the viewer is not optimized for highly complex PTM-information and DAS-enabled resources of PTMs are limited, thereby limiting annotations available on this viewer. Ideally, future versions of ProteomeScout will incorporate a DAS protocol for improved access to PTM-information for DAS-based services and conversely utilize more DAS-based protocols for retrieval of external database information.

It is the growth from PTM- to protein-centric features that led us to rename our original resource from PTMScout to ProteomeScout. All the functionality of PTMScout (7) has been incorporated into ProteomeScout and, due to the loss of support in PTMScout-dependent resources, PTMScout now redirects to ProteomeScout. We feel that this expansion in protein-centric resources, as well as dataset ownership features, will make ProteomeScout useful for a range of molecular and biological researchers: from those interested in particular aspects of individual proteins, to researchers wishing to analyze proteome-wide trends and to researchers making high-throughput measurements of post-translational modifications.
